# Use of Electroanatomic Mapping Systems beyond Electrophysiologic Studies

**DOI:** 10.1016/s0972-6292(16)30652-0

**Published:** 2013-08-01

**Authors:** Serkan Cay

**Affiliations:** Department of Cardiology, Division of Arrhythmia and Electrophysiology, Yuksek Ihtisas Heart-Education and Research Hospital, Ankara, Turkey

**Keywords:** Electroanatomic Mapping

I have read with great interest the case presentation entitled 'Utility of the NavX® Electroanatomic Mapping System for Permanent Pacemaker Implantation in a Pregnant Patient with Chagas Disease' by Velasco et al. in a recent issue of the journal [1]. The authors have presented a pregnant with Chagas disease and complete AV block requiring pacemaker implantation and treated with a DDD mode pacemaker implanted using a 3-D electroanatomic mapping system, EnSite NavX®. They have stated that pacemaker implantation using electroanatomic mapping systems can be the first choice in pregnant patients to avoid radiation exposure. Some important issues should be mentioned; first, the gestational age of the fetus of the presented female was 31 weeks. Beyond the 20th week of gestation, the fetus becomes more resistant to hazardous effects of radiation because its developmental process is completed. Therefore, the fetus gains resistance to adverse effects of radiation similar to that of a newborn. There is no absolute risk for developmental defects or miscarriage from diagnostic examinations at later stages of pregnancy. Until present, there is no data about an elevated fetal risk of congenital anomalies, intellectual disability, growth retardation, or pregnancy loss at doses of radiation exposure to the pregnant female of <50 mGy. During a radiofrequency catheter ablation, fetal exposure dose is approximately 3 mGy. [2]. All preventive measures should be taken to avoid radiation exposure for all pregnant women although there are data regarding resistance to hazardous effects of radiation. Lead or antimony aprons or shields over abdominal region can be used with short bursts of fluoroscopy as in the case. Second, the authors have stated that electroanatomic shape of the right heart including the superior vena cava, right atrium, tricuspid annulus, right ventricular apex and right ventricular outflow tract had been drawn although they have presented a limited shape. The ventricular lead was placed in the outflow tract and the atrial lead in the right atrial appendage. P-wave amplitude was good and R-wave amplitude was acceptable. R-wave amplitude could be better if the active fixation lead was tried to place in a region with a good intracardiac signal during electroanatomic mapping. Lastly, teleradiographic position of the ventricular lead obtained after delivery seems different from initial positioning. It has been located at the bottom of the right ventricle near the apex but far from the outflow tract.
